# Biogeography of *Paenibacillus larvae*, the causative agent of American foulbrood, using a new multilocus sequence typing scheme

**DOI:** 10.1111/1462-2920.12625

**Published:** 2014-11-28

**Authors:** Barbara J Morrissey, Thorunn Helgason, Lena Poppinga, Anne Fünfhaus, Elke Genersch, Giles E Budge

**Affiliations:** 1 Biology Department, University of YorkPO Box 373, York, YO10 5YW, UK; 2 Food and Environment Research AgencySand Hutton, York, YO41 1LZ, UK; 3 Institute for Bee ResearchFiedrich-Engels-Str. 32, Hohen Neuendorf, 16540, Germany; 4 Institute of Microbiology and Epizootics, Freie Universität BerlinRobert-von-Ostertag-Str. 7–13, Berlin, 14163, Germany

## Abstract

American foulbrood is the most destructive brood disease of honeybees (*Apis mellifera*) globally. The absence of a repeatable, universal typing scheme for the causative bacterium *Paenibacillus larvae* has restricted our understanding of disease epidemiology. We have created the first multilocus sequence typing scheme (MLST) for *P. larvae*, which largely confirms the previous enterobacterial repetitive intergenic consensus (ERIC)–polymerase chain reaction (PCR)-based typing scheme's divisions while providing added resolution and improved repeatability. We have used the new scheme to determine the distribution and biogeography of 294 samples of *P. larvae* from across six continents. We found that of the two most epidemiologically important ERIC types, ERIC I was more diverse than ERIC II. Analysis of the fixation index (F_ST_) by distance suggested a significant relationship between genetic and geographic distance, suggesting that population structure exists in populations of *P. larvae*. Interestingly, this effect was only observed within the native range of the host and was absent in areas where international trade has moved honeybees and their disease. Correspondence analysis demonstrated similar sequence type (ST) distributions between native and non-native countries and that ERIC I and II STs mainly have differing distributions. The new typing scheme facilitates epidemiological study of this costly disease of a key pollinator.

## Introduction

*Paenibacillus larvae*, a Gram-positive spore-forming bacterium, causes American foulbrood (AFB), which is the most destructive brood disease of the honeybee (*Apis mellifera*). AFB is contagious because of the large numbers of highly resistant spores that are produced and efficiently transmitted by contaminated adult bees within and between colonies (Lindström, 2008; Lindström *et al*., 2008a,b[Bibr b43],[Bibr b44]). Only the spores are infective and are fed to bee brood by nurse bees in contaminated larval food (glandular secretions and processed honey) (Yue *et al*., 2008). Once infected, larvae usually die within 3–12 days (Genersch *et al*., 2005; Rauch *et al*., 2009). *Paenibacillus larvae* spores are able to remain infective for more than 35 years in old hives and are resistant to extremes of temperature (Hasemann, 1961). This makes the control of the disease difficult because human activity can spread the disease over long distances and previously dormant strains may cause an outbreak several years after the original outbreak.

Antibiotics only affect the vegetative stage of the bacterium, masking the symptoms of AFB; they have no effect on the infective spores (Genersch and Otten, 2003). In many countries, burning infected colonies and hive materials is thought to be the most effective way of preventing the spread of AFB. Therefore, whether AFB is ignored or treated, the colony will be killed, which leads to considerable economic loss to global apiculture.

AFB is found on every continent where honeybees are kept (Matheson, 1993). The disease is spread by both humans and bees, and it is spread predominantly via horizontal routes although it has been shown to spread vertically (Fries *et al*., 2006; Lindström *et al*., 2008a). Horizontal transmission of AFB can occur by several means because of humans through the movement of contaminated honey or the movement of contaminated hives or equipment (Genersch, 2010). AFB can also be spread horizontally by bees, either through the movement of adult bees between colonies (drifting) or the behaviour of foragers (robbing) (Lindström *et al*., 2008a). Using genetic markers to identify outbreaks caused by closely related strains can give important evidence to confirm the source and routes of disease transmission. The shortcomings of phenotypically based typing methods for *P. larvae* (Genersch *et al*., 2006) have led to the development of molecular typing methods based on the microbial DNA sequence (Alippi and Aguilar, 1998; Genersch and Otten, 2003; Genersch *et al*., 2006; Antúnez *et al*., 2007; Pentikäinen *et al*., 2008). Four genotypes of *P. larvae* have been identified based on repetitive-element polymerase chain reaction (PCR) (rep-PCR) using enterobacterial repetitive intergenic consensus (ERIC) primers (Genersch *et al*., 2006). These four genotypes were shown to form two clusters using pulsed-field gel electrophoresis (PFGE) (Genersch *et al*., 2006). ERIC types differ in phenotype including morphology (Genersch *et al*., 2006), metabolic capacity (Neuendorf *et al*., 2004) virulence (Genersch *et al*., 2005; Rauch *et al*., 2009) and virulence factors (Poppinga *et al*., 2012; Fünfhaus *et al*., 2013). The above typing schemes have been used to categorize crude patterns of *P. larvae* distribution in Europe (Genersch and Otten, 2003; Pentikäinen *et al*., 2008; Loncaric *et al*., 2009; Di Pinto *et al*., 2011), the Americas (Alippi *et al*., 2004; Antúnez *et al*., 2007) and Australasia (Alippi *et al*., 2004). However, rep-PCR methods have the disadvantage that they are difficult to repeat or to standardize between laboratories and therefore comparisons between different studies is difficult (Rusenova *et al*., 2013). Recently, it was shown that the four ERIC genotypes can also be discriminated via matrix-assisted laser desorption/ionization time-of-flight mass spectrometry (Schäfer *et al*., 2014), providing a cost-effective, reliable and highly reproducible alternative tool for *P. larvae* ERIC typing. The advantage of the established ERIC scheme for *P. larvae* (Genersch *et al*., 2006) is that it allows grouping into biologically relevant genotypes differing in practically important phenotypic features. However, it does not give enough resolution to be used effectively as an epidemiological tool to study disease spread. Therefore, a state-of-the-art method providing sufficient resolution to be informative to epidemiological studies is urgently needed in order to enhance differentiation beyond the four ERIC genotypes.

The utilization of sequence-based typing would allow a single, universal and unambiguous scheme that would help us to better understand the global spread of this damaging disease. Multilocus sequence typing (MLST) can provide a standardized set of strain types that can be used to study bacterial population structure and evolution at both a global and a more local scale. MLST is based on unambiguous DNA sequences and allelic profiles can be shared between laboratories using online databases such as PubMLST (Jolley and Maiden, 2010). MLST schemes have become the gold standard for epidemiological studies, providing insight into the epidemiology of human pathogens such as the *Bacillus cereus* group (Helgason *et al*., 2004) to which *P. larvae* is related. Generally, MLST schemes consist of short regions of six or seven housekeeping genes that evolve at a slow even pace across all strains (Maiden, 2006).

Here, we report the development of a novel seven genes MLST scheme to enhance differentiation within the species *P. larvae* and we use this scheme to identify global patterns in the population structure of *P. larvae*.

## Results

### Loci discovery and primer design

In total, 31 primer pairs, including non-coding loci, were tested against *P. larvae* isolates representing all four ERIC types. The majority of loci were rejected because of low diversity between test isolates (Table S1). Of the remaining loci, seven offered the largest diversity within the 294 isolates of *P. larvae* tested: *clpC* (catabolite control protein A), *ftsA* (cell division protein), *glpF* (glycerol uptake facilitator protein), *glpT* (glycerol-3-phosphate permease), *Natrans* (forward sodium dependant transporter), *sigF* (sporulation sigma factor F) and *rpoB* (RNA polymerase beta subunit) (Table 1).

**Table 1:** MLST primer sequences

Gene	Forward primer	Reverse primer	Annealing temperature (°C)
*clpC*	5′TTTGGAAGATTTACTGAACGA3′	5′ATCAGAACCGGGTTATTTTT3′	52
*ftsA*	5′AAATCGGTGAGGAAGACATT3′	5′TGCCAATACGGTTTACTTTA3′	52
*glpF*	5′GTCAGCGGGGCTCATTTA3′	5′TGCTTACGATGAGAAATCCT3′	52
*glpT*	5′GGATTGAAAAACTTGAAACG3′	5′CATGCTGAGAGAAATCTTCC3′	52
*Natrans*	5′GCTTCGGTAATGGTAACCTA3′	5′TTGAACCCATTGTAAATTCC3′	52
*rpoB*	5′ATAACGCGAGACATTCCTAA3′	5′GAACGGCATATCTTCTTCAG3′	52
*sigF*	5′GTCACTGAAGGAATTGGCTA3′	5′TATCTGGTTACGGATGGACT3′	52

Fragment length and G + C content for the seven selected loci ranged from 271 bp (*glpF*) to 541 bp (*clpC*) and 43.8% to 48.7% respectively (Table 2). The percentage of variable sites ranged from 0.65 (*ftsA*) to 2.0 (*Natrans*) resulting in 4–6 alleles per locus (Table 2). The d_N_/d_S_ values were all lower than 1 for all genes, except *glpF*, which contained a deletion. The ratio of non-synonymous and synonymous substitutions (*d_N_/d_s_*) measures the level of selection in a protein-coding gene. To ensure consistency in an MLST, it is preferable that each locus is under stabilizing selection. However, genes that are under positive selection may give more resolution to the scheme (Maiden, 2006). The ratio of *d_N_/d_S_* indicates purifying selection (negative selection) if values are < 1, positive selection if values are > 1 and neutral evolution if values are close to 1. A value approaching 1 may also indicate a combination of positive and purifying selection.

**Table 2:** Feature summary of seven loci selected for *P. larvae* typing scheme

Locus	Sequence length (bp)^a^	Number of alleles	Mean G + C^b^ content	Number of polymorphic sites^a^	Number of non-synonymous substitutions	*d_N_/d_S_* ratio
*clpC*	541	5	48.7	4(0.74)	3	0.4183
*ftsA*	464	4	46.0	3(0.65)	2	0.7756
*glpF*	271	6	45.2	5(1.85)	4	1.4835
*glpT*	502	4	47.4	9(1.80)	6	0.5569
*Natrans*	490	6	46.8	10(2.00)	6	0.2180
*rpoB*	339	5	48.7	4(1.50)	2	0.4276
*sigF*	345	4	43.8	4(1.20)	1	0.1607

a Percentage of polymorphic sites in parentheses.

b Guanine-cytosine content.

The index of association (I_A_) was significantly different from 0 when only one representative of each sequence type (ST) was included in the computation (1.16; *P* = 0.000), indicating limited recombination events and a clonal populations structure in *P. larvae*. The I_A_ measures the extent of linkage equilibrium within a population by quantifying the amount of recombination among a set of sequences and detecting association between alleles at different loci (Smith *et al*., 2014).

**Fig 1:**
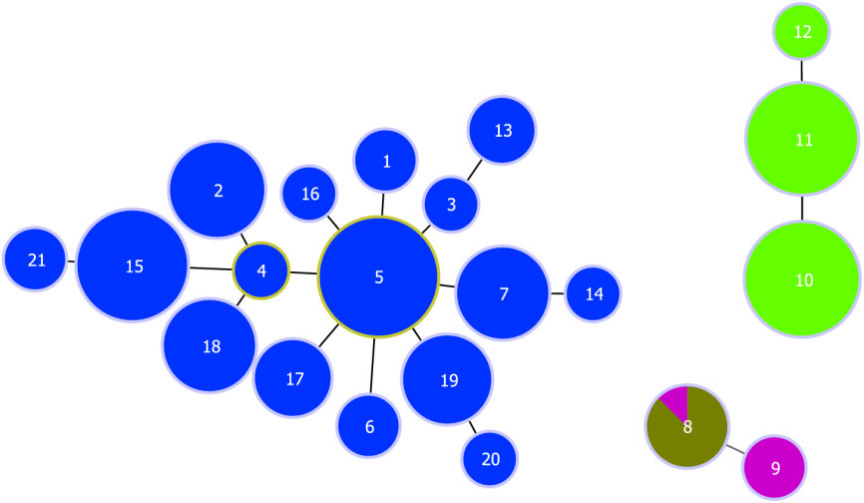
eburst diagram of *P. larvae* MLST scheme. The numbers in the circles represent the ST of the isolates (Table 2). The size of the circle represents the number of isolates with that type (Table 2). STs with variation at more than three loci are not connected. Clockwise from left, groups are: isolates typed as ERIC I, isolates typed as ERIC II and isolates typed as ERIC III and IV.

**Fig 2:**
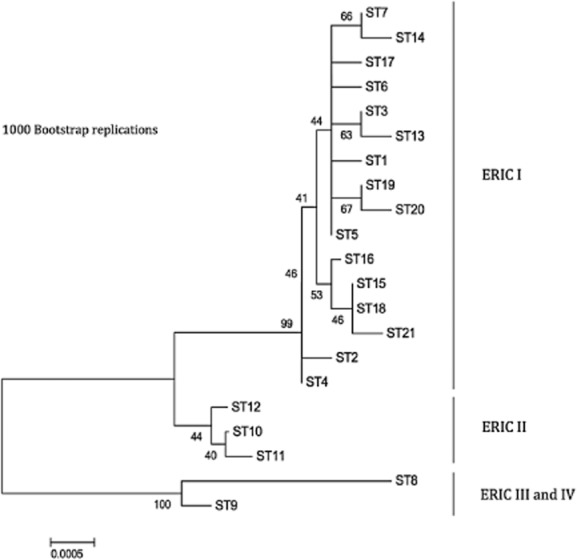
Neighbour-joining tree of all sequence types. The evolutionary history was inferred using the neighbour-joining method. The optimal tree with the sum of branch length = 0.01386545 is shown. The percentage of replicate trees in which the associated taxa clustered together in the bootstrap test (1000 replicates) is shown next to the branches. The tree is drawn to scale, with branch lengths in the same units as those of the evolutionary distances used to infer the phylogenetic tree. The evolutionary distances were computed using the Maximum Composite Likelihood method and are in the units of the number of base substitutions per site.

**Fig 3:**
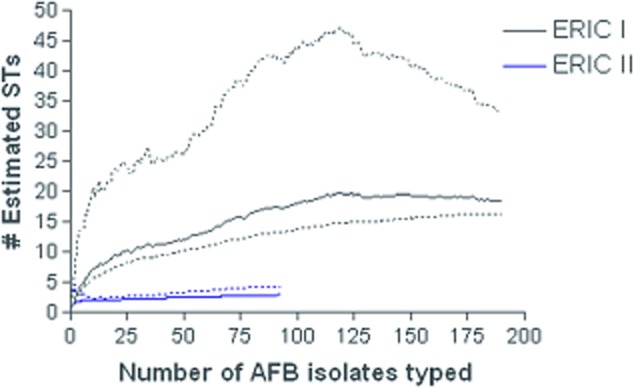
Chaol estimates of ERIC I and ERIC II ST richness as a function of sample size. Dotted lines are 95% CIs and were calculated with the variance formula derived by Chao (1987). The lower solid line represents ERIC II and the upper solid line represents ERIC I.

Typed isolates included 173 ERIC type I, 92 ERIC type II, 3 ERIC type III and 7 ERIC type IV and 19 isolates where ERIC type was not determined because of a shortage of DNA (Table S2). At least one isolate of each ST was ERIC typed. The 7-gene MLST scheme resolved the 294 *P*. *larvae* isolates into 21 different STs (Fig. 1 and Fig. S2). The allele sequences have been submitted to the EMBL database under Accession numbers HG530076 to HG530109. The entire scheme is available at http://pubMLST.org/plarvae/ (Jolley and Maiden, 2010). ST designations represented a single ERIC grouping except for a single ERIC III isolate that grouped with ST8 (Figs 1 and 2). Isolates from ERIC I were separated into 16 STs, whereas ERIC II isolates were only separated into three separate STs. The Chao1 estimates suggest this difference in observed diversity was unlikely to be due to a biased sampling effort. After an initial increase, the mean Chao1 estimate for all geographical regions became relativley level as sample size increased (Fig. 3), therefore we compared the ST diversity estimates at the highest sample size for each ERIC type (Hughes *et al*., 2001). Total diversity of ERIC types is significantly different as estimated by Chao1 (Fig. 3). Chao1 estimates that ERIC I has 18.49 STs [95% confidence intervals (CIs) 16.37 and 32.91], and ERIC II has 3 STs (CIs 3 and 4.49). The STs were roughly split following the pattern of the ERIC typing scheme with different ERIC I and II types forming distinct groups with both the eburst and phylogenetic analysis (Figs 1 and 2; Table S2). ERIC III and IV isolates were less distinct with one isolate (P6266) LMG 16252) ERIC typed as ERIC III but sequence typing as ST8 and grouping with ERIC IV isolates (Fig. 1, Table S2). All isolates within ERIC I and II are linked by single loci variants, whereas the ERIC III and ERIC IV STs differ from each other at two loci. ERIC I and ERIC II isolates differ from one another by at least four loci (link not shown in Fig. 1) and ERIC III isolates differ from ERIC I isolates by at least six loci (Fig. 1). The ERIC III ST 9 is made up of only two isolates found in Chile (Table S2).

### Population structure and biogeography

The two most common and widespread types of ERIC I were ST 15 and 5, which were each found causing disease in multiple countries and across five continents (Table S2). Contrastingly of the two most common STs of ERIC II, ST10 was well distributed and ST 11 was found only in Germany and countries to the east of Germany. In this study, no ERIC II isolates were collected from countries to the west of Germany within the native honeybee range.

**Fig 4:**
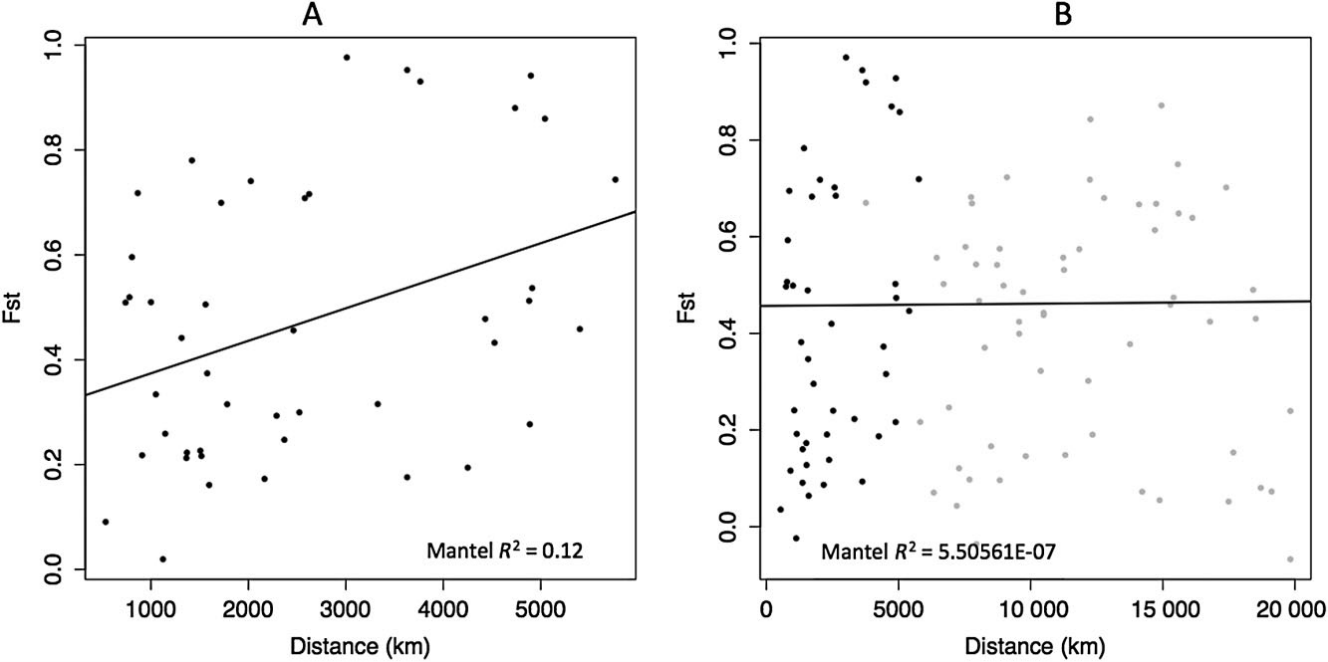
F_ST_ by distance.A. Describes the F_ST_ by distance of *P. larvae* populations within the native range of the host (*Apis mellifera*).B. Describes the F_ST_ by distance of *P. larvae* populations both within (dark grey dots) and outside of the native range of the host (light grey dots).

The relationship between fixation index (F_ST_) and geographic distance of the global populations of *P. larvae* were not significant (*P* = 0.996, *r*^2^ = 5.506 × 10^−7^), suggesting no relationship between genetic and geographic distance when considering the sampled global population of *P. larvae*. However, when the analysis was restricted to isolates collected from within the native range of honeybees [Europe, Africa and Eastern Asia; 226 of 294 isolates (see Table S2)] a significant relationship between genetic and geographic distance was detected (*P* = 0.01, *r*^2^ = 0.122; Fig. 4).

### Correspondence analysis (CA)

**Fig 5:**
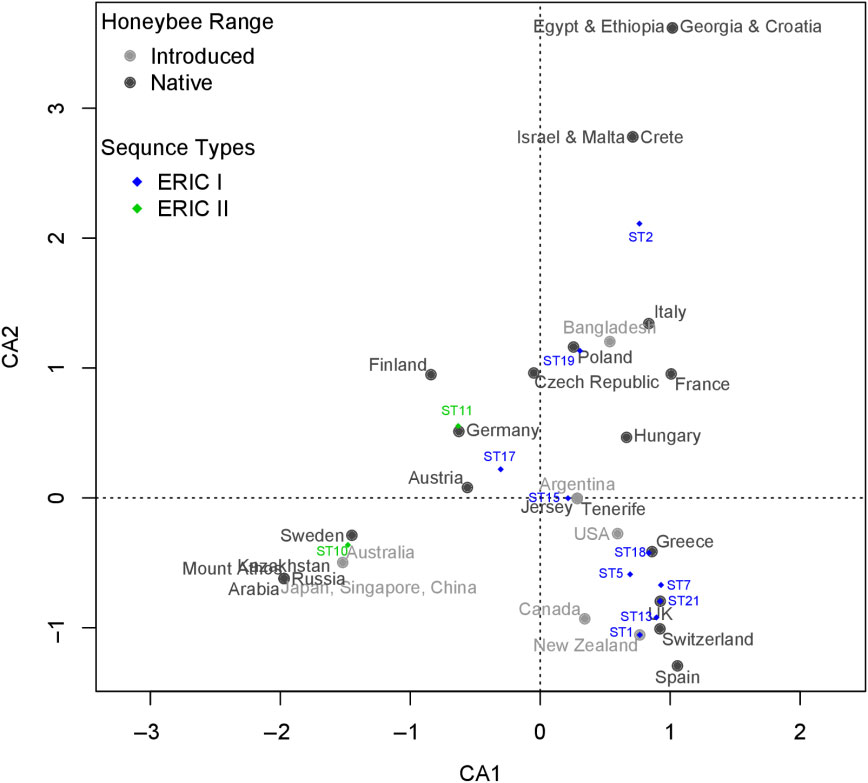
Correspondence analysis ordination graph. The CA ordination graph illustrates the associations among countries of isolate origins and MLST STs. Filled circles represent countries (dark grey represents native host range, light grey represents introduced host range) Diamonds represent STs.

The ordination graph (Fig. 5) describing the results of the CA shows a clear split in the distribution of the two ERIC types. In addition, Fig. 5 shows no split in the distribution of countries, whether they were in the native range of honeybees or not. This suggests that most STs are found in both the native range and the countries outside of the native range.

The proportion of variance explained by the first two eigenvectors was 0.4026. In the ordination graph describing the results of the CA (Fig. 5), STs that have similar distribution are represented by points closer together in space and the proximity of STs to countries indicate that those STs are associated with that country. The first axis highlights the difference in distribution between ERIC I STs and ERIC II STs. The ERIC II STs (ST 10 and ST 11) cluster on the left (negative values) of CA1, and the ERIC I STs on the right, with the exception of ST17. ST10 groups with Arabia, Mount Athos, Kazakhstan, Russia, Sweden and Australia as well as mixed origin isolates from China, Singapore and Japan. ST11 is associated with Germany and Finland. Native range designation did not appear to influence the distribution of the points on the ordination plot, i.e. countries where honeybees have been introduced grouped with countries where honeybees are native.

### Rarefaction

Rarefaction curves comparing the sampling effort for each continent show steep curves for all except Europe. However, when only data from Europe were analysed at country level, it was clear that only Germany and the UK were sampled to adequately describe the resident ST populations. This suggests that further sampling in other countries would yield previously unidentified STs.

## Discussion

AFB is a serious disease of honeybee brood with near global distribution; however, disease epidemiology is poorly understood, in part due to an absence of a repeatable method to discriminate between strain types. We have developed the first MLST scheme, which ratifies and extends the established four-group ERIC typing scheme. The new scheme describes 21 different *P. larvae* STs, therefore providing improved resolution when compared with ERIC typing. Although other typing schemes, such as PFGE (Pentikäinen *et al*., 2008) offer greater resolution, i.e. more types, than MLST, these methods are difficult to repeat, preventing comparisons between published studies. MLST is now the gold standard for epidemiological studies (Francisco *et al*., 2012) and results can be applied to derive epidemiological meaning both locally and internationally.

Our MLST scheme was able to identify a significant relationship between geographic distance and genetic distance among *P. larvae* populations within the native range of the honeybee (Fig. 4A). This relationship is surprising given the history of human movement of bees within Europe in particular (De la Rúa *et al*., 2009). It might be expected that because of past honeybee movements, mixing of *P. larvae* STs might be equally distributed throughout the native range; however, this is not the case. A detectable link between physical and genetic distance remains despite these large-scale movements and endemic populations of *P. larvae* still appear to exist within the native range. Such a result could suggest that certain *P. larvae* strains are adapted to local honeybee populations. Honeybees are known to differ in their ability to resist pathogens (Jensen *et al*., 2009; Büchler *et al*., 2010), and AFB-tolerant honeybees have been bred in Argentina (Spivak and Reuter, 2001). However, little is known more generally about the susceptibility of honeybee races to different strains of *P. larvae*. A more comprehensive understanding of inter-race susceptibility of *A. mellifera* is required to understand whether host factors are in part responsible for the significant pathogen population structure within the native range of *A. mellifera*.

The commercial interest in honeybee hive products has lead to the spread of *A. mellifera* far beyond its native geographic range. This global industry has facilitated the spread of honeybee pests and pathogens such as the ectoparastic mite *Varroa destructor* (Solignac *et al*., 2005), *Nosema ceranae*. (Klee *et al*., 2007) and Israel acute paralysis virus (Palacios *et al*., 2008) and it is known that *P. larvae* spores can remain infective in honey (Morse, 1992; Govan *et al*., 1999; Hansen *et al*., 2003). Our data indicate that when AFB has been moved outside the native honeybee range, any evidence of significant pathogen population structure breaks down (Fig. 4B). This finding suggests that the international trade in honeybees and their hive products may have moved *P. larvae* multiple times in a non-systematic manner to infect honeybees beyond the native range. Despite haphazard movements of host and pathogen, there remain some interesting links between historic honeybee movements and ordination graph observations linking STs to locations (Fig. 5). Some historic honeybee movements are recorded in the literature, and it is possible to trace, for example, honeybee imports into USA and New Zealand back over 400 years to their origins in Europe (Donovan, 1980; Goulson, 2003). It is perhaps unsurprising that the CA (Fig. 5) groups countries together from within and out with the native host range group, given that there are traceable links and potential disease transmission routes between these countries (know import/exports of bees and hive materials).

Novel types were sometimes identified only outside the native range of *A. mellifera* with ST3 only found in New Zealand and ST4, ST9, ST16 only identified in the Americas (Table S2). There are two plausible explanations for this observation. First, the presence of unique types outside the native range could suggest that these types have evolved since becoming isolated from the founder pathogen population. Second and perhaps more likely, our sampling scheme was more intensive in some countries (such as the UK and Germany) and superficial in other countries within the native range (Table S2). Therefore, it is likely that our sampling scheme was not sufficiently exhaustive to detect the full extent of ST diversity within the native range of *A. mellifera*.

*Paenibacillus larvae* isolates classed as ERIC I were more diverse than those classed as ERIC II, containing 16 unique STs compared with only 3 for ERIC II (Fig. 1). The results of the Choa1 estimate (Fig. 3) show that although the sampling was uneven with more ERIC I isolates typed than ERIC II, for these data, the Chao1 estimator levels off (Fig. 3), suggesting that the Chao1 estimate is relatively independent of sample size. ERIC I and II isolates differ phenotypically in many ways for example endospore resistance to temperature (Forsgren *et al*., 2008), rates of sporulation (Saville, 2011), time to host death (Genersch *et al*., 2005; 2006; Rauch *et al*., 2009) and it was suggested that they employ different strategies for killing larvae (Poppinga *et al*., 2012; Fünfhaus *et al*., 2013; Djukic *et al*., 2014). Perhaps these differences account for the disparity in diversity, as some STs may be better able to spread to new areas.

It had previously been assumed that ERIC II is confined to Europe and that it is not rare in Germany or Austria (Genersch, 2010). However, our data suggest that ERIC II STs are much more widely distributed than previously thought, being present in Asia, North America and Australasia as well as Europe, although in this study, no ERIC II isolates were found in Europe in countries west of Germany. However, there is evidence that ERIC II is present in Germany in areas that border France and Belgium (Saarland, see Table S2), which might suggest its presence in these countries. This study has split ERIC II into three new STs (Fig. 1) with ST10 and ST11 being common and ST12 being identified once. Considering the prevalence of ST10, it seems unusual that ST11 remains localized. These differences in prevalence and distribution between ERIC II STs could reflect a difference in phenotype, rather than an artefact of sampling bias, as there was no previous method to discern among ERIC II strains. Future work could include genomic and phenotypic comparisons between ERIC II STs, to identify reasons for the observed differences in distribution.

Interestingly, ERIC I and II have mainly different distributions as indicated in the ordination graph, ST17 is the only ERIC I isolate on the left side of the graph (Fig. 5). No ERIC II STs (ST10, ST11) were found in Africa or in the west of Europe in the native range of *A. mellifera* (Table S2). The fact that they were not found in Africa and much of the west of Europe may be explained by poor sampling effort. However, the UK was more thoroughly sampled and still no instances of ERIC II STs were found. One suggestion for their differing distribution might be that ERIC II STs are host specific. There are around a dozen subspecies of *Apis mellifera*, and they are split into four groups M, W, C and O (African, Western Europe, Eastern Europe and Western Asia respectively). It is thought that *Apis mellifera* originated in Africa, and from there, it is likely that there was one expansion into western Europe and either one or two expansions into the east (eastern Europe and Asia) (Whitfield *et al*., 2006). This means that there is a closer relationship between the western European group of subspecies and African subspecies than between eastern and western European subspecies. This may explain the split in the distribution of ERIC types with STs of ERIC II only being found in Germany and countries to the east within Europe and Asia. The C group subspecies *Apis mellifera carnica* has almost completely replaced the W group *Apis mellifera mellifera* in Central European countries such as Germany, whereas in Poland, where no ERIC II was found, the majority of bees are still *A. m. mellifer*a (Meixner *et al*., 2007). The native range of *A. m. mellifera* is from the UK to Scandinavia and from France to Poland. However, Italian (C group) and Carnelian (*carnica*) bees have been transported around Europe in the *A. m. mellifera* range and hybridization has occurred; in fact, it is thought that in Scandinavia and the UK, the bees are a mixture of all three subspecies (De La Rúa *et al*., 2001; Jensen *et al*., 2005) and although both Scandinavia and the UK are thought to have a similar mix of subspecies, ERIC II STs were found in Scandinavia. As we have no information on the host subspecies in our data set, it is impossible to determine whether different *P. larvae* STs affect *A. mellifera* subspecies differently. Future work could involve testing this theory by infecting larvae of the honeybee subspecies with a range of STs of *P. larvae*.

Although local epidemiological observations were not the primary purpose of this study, our data offer some evidence for the hitherto unknown origin of the 2010 AFB outbreak in Jersey, an island in the English Channel. Our scheme matched one Jersey isolate (Table S2) with a ST that was only found in France (ST6), Jersey's closest neighbour, providing evidence of a potential transmission route and demonstrating the potential power of MLST to inform disease aetiology when coupled to more extensive local sampling efforts.

In summary, we have developed an important new tool for describing the genetic structure of *P. larvae*, which raises unanswered questions about differential host susceptibilities to *P. larvae* STs. Future proofing in an age of rapid advancement in sequencing technologies is an important consideration, and MLST is compatible with methods that could potentially supersede, such as whole genome sequencing (Larsen *et al*., 2012). National laboratories responsible for the control of AFB can now use this scheme to gather comprehensive data on ST locations and expand the online database http://pubMLST.org/plarvae/ (Jolley and Maiden, 2010) to build a comprehensive multinational data set to better understand the distribution and transmission networks of AFB on a global scale. Our scheme therefore provides the first universal method for the description of strains of *P. larvae* and will increase our understanding of the epidemiology of this damaging and costly disease at many spatial scales.

## Experimental procedures

### MLST development

Whole genome comparisons were made between two genetically diverse strains of *P. larvae* (Genersch *et al*., 2006) (p6678 and p6993; LMG 16241 and LMG 16247, ERIC I and ERIC IV respectively). Housekeeping genes used in a previous typing scheme for the related *B. cereus* group (Helgason *et al*., 2004) were found to have no variation when compared between these two isolates, so novel regions with 80–90% similarity were identified. Pairwise comparisons were made using the online program doubleact and the result visualized using Artemis Comparison Tool (Carver *et al*., 2005) and mega version 5 (Tamura *et al*., 2011).

Two further genomes were later compared [DSM25719 (ERIC I), NCBI acc. No. ADFW01000002; DSM25430 (ERIC II), NCBI acc. No. NC_023134]. This led to the discovery of additional suitable loci, which were identified using the *B. cereus* Group Typing Database (University of Oslo, 2012) and by targeting genes likely involved in observed phenotypic differences in sporulation frequency between ERIC types (Saville, 2011).

### Primer design

Primers were designed to candidate loci using primer 3 (v0.4.0) (Rozen and Skaletsky, 2000), and oligocalc (Kibbe, 2007) with an optimum melting temperature of 55°C (± 5°C). In total, 31 primer pairs were tested including those for non-coding loci to give a scheme composed of seven coding loci. The primer sets were used to amplify a panel of *P. larvae* isolates of all four ERIC types. Any primer set that did not add extra resolution to the scheme was rejected (Table S1).

The PCR reaction conditions were as follows: After the initial activation step (3 min, 95°C), 35 cycles at 95°C for 30 s, 52 °C for 30 s and 72°C for 1 min were run followed by a final elongation step at 72°C for 10 min.

### Global isolates

In total, 294 *P*. *larvae* isolates from existing national and international culture collections were recovered on either blood agar or brain heart infusion agar and PLA (de Graaf *et al*., 2013). Briefly, spore containing honey was either heated at 90°C for 6 min prior to plating (Genersch and Otten, 2003) or plated directly without heat treatment (Forsgren *et al*., 2008) because temperatures above 90°C or an incubation time of 10 min could have negatively affected germination of ERIC II strains (Forsgren *et al*., 2008). Isolates were collected from 38 countries from across six continents: Africa (*n* = 5), Australasia (*n* = 26), Asia (*n* = 27), Europe (*n* = 199), North America (*n* = 16) and South America (*n* = 4) as well as some of mixed origin and some from culture collections (*n* = 17) (Table S2).

DNA was extracted from *P. larvae* cultures using a simple Chelex method. Bacteria were transferred to 300 μl 6% Chelex®100 and heated to 56°C for 20 min followed by boiling for 8 min. DNA extracts were stored at −20°C until required.

ERIC typing was completed using the method described in Genersch and colleagues (2006).

### Sequencing

PCR products were purified using Qiagen® PCR purification and sequenced on the ABI 3730xl 96-capillary DNA Analysers. Sequences were aligned using clustalw in mega version 5 (Tamura *et al*., 2011) and allele types were counted and numbered in order of discovery as described by Aanensen and Spratt (2005). An allele was identified as a sequence or sequences with one or more genuine nucleotide difference from previously assigned sequences. The combination of allele numbers for each of the target genes gives the allelic profile or ST of an isolate. All putative loci were amplified from a panel of *P. larvae* isolates representing all four ERIC types. Loci that failed to add resolution to the scheme were rejected, and all isolates were typed using the final MLST scheme.

### MLST analysis

The ratio of non-synonymous and synonymous substitutions (*d_N_/d_s_*) of MLST gene fragments was determined using the modified Nei–Gojobori method (Nei and Gojobori, 1986) in the program start2 (Jolley *et al*., 2001).

Recombination was tested using the I_A_ with the program start2 (Jolley *et al*., 2001).

### Population structure and biogeography

phyloviz (Francisco *et al*., 2012) was used to analyse allelic profiles using the goeburst algorithm (Feil *et al*., 2004; Francisco *et al*., 2009). The program was used to discover clonal complexes and infer founder clones (Francisco *et al*., 2009). The most parsimonious patterns of descent of all isolates in each clonal complex from the predicted founder(s) were calculated as previously described (Francisco *et al*., 2009; 2012[Bibr b18],[Bibr b19]). A phylogeny was also constructed from an alignment of 2948 sites representing the concatenated sequences of each ST. The sequences were aligned using clustalw, as implemented in mega 5.2 (Tamura *et al*., 2011). The phylogenetic analysis was then carried out using the neighbour-joining method and the Maximum Composite Likelihood model as implemented in mega 5.2 (1000 replications).

To test the population structure of *P. larvae* among different countries, pairwise FST was calculated using the haploDiv command in the r package diversity (Keenan *et al*., 2013) and bootstrapped 95% CIs (500 repeats) were calculated. The 40 single nucleotide polymorphisms (SNPs) identified in the concatenated MLST gene sequences were used to derive pairwise F_ST_ between populations. Populations were taken as all samples from a single country, see Table S2. Countries where there were samples from fewer than five isolates were discounted (see Table S2) or grouped: Samples from Bangladesh, Japan, China, Singapore and Mongolia became Asia. Geographic Distances were taken as the great circle distance between the centre point location for each country or group of countries. A Mantel test (Mantel, 1967) with 1000 replications was used to determine whether the correlation between physical distance and F_ST_ was significantly different from a random sample of the data. All results were visualized using r (version 2.15.2) (R Core Team, 2012).

CA was also applied to the data. The CA takes into account the STs present in each country to investigate associations between STs (which types were commonly found together) and patterns in their distribution (which countries are associated with the STs).

Finally, rarefaction curves were constructed to compare the sampling efforts between different continents and between countries within Europe. r library vegan (Oksanen *et al*., 2013) was used to carry out these final two analyses.

## Supporting information

**Table S1.** Genes for which primers were designed and then rejected.

**Table S2.** Origins of isolates typed with MLST scheme.
