# Supplementary material for: Biogeography of Paenibacillus larvae, the causative agent of American foulbrood, using a new multilocus sequence typing scheme
Source: Environ Microbiol. 2014 Nov 28;17(4):1414–24. doi: 10.1111/1462-2920.12625 (PMC4405054; doi:10.1111/1462-2920.12625)
Supplement: Supplementary file 1 — Table S1. Genes for which primers were designed and then rejected. [file emi0017-1414-sd1.pdf]

Table S1. Genes for which primers were designed and then rejected

Gene	Forward primer	Reverse primer	Annealing temp (°C)
adk	5'ATGCCTACCTTGCCTAACAT3'	5'ATGCTTCTCCGTTTCGTG3'	**56**
ccpA	5'GTTTCTCGGGTTGTGAATAA3'	5'CCGTCTACCTGTTTTTCAAG3'	**55**
chiA	5'GCGGATGAACTCTAGTGAAC3'	5'ACGATTAAAACGAGCGAAC3'	**55**
gdh	5'TATCATTACAGGGGGTTCC3'	5'CATAGCTAATACGCCTGCTT3'	**55**
gmk	5'AAAGACAATGGAAAGAGAAAGA3'	5'ATTGTTCCATCAGACGAATC3'<br>5'CTGATGGAACAATATGATTACG3'	**55**
ilvD	5'GATGAACTGTCTGGCTGAAG3'<br>5'AAGGCGGCATCATAAAAGTC3'	5'CCCTGTTCATTATGTGGATTAT3'<br>5'AATTCTTCTTCGGATATTTGC3'	**57**<br>**56**
panC	5'TCTCTCCCCAATGAAATATG3'<br>5'CTTTATCCGGCTGAACAATA3'	5'TTAAAGATGCACAGCAAGTG3'<br>5'GGAATTAAAAACGGGACATA3'	**55**<br>**55**
sigK	5'GGAGAAGACCTGGAAGATTT3'	5'TTTTGTAAAATTCATGATAAAGC3'	**54**
tpi	5'ATATATTCGCGCACGTTTC3'	5'ACGTTCAAGGGAACCTCTAT3'	**55**
