# Supplementary material for: Biogeography of Paenibacillus larvae, the causative agent of American foulbrood, using a new multilocus sequence typing scheme
Source: Environ Microbiol. 2014 Nov 28;17(4):1414–24. doi: 10.1111/1462-2920.12625 (PMC4405054; doi:10.1111/1462-2920.12625)
Supplement: Supplementary file 2 — Table S2. Origins of isolates typed with MLST scheme. [file emi0017-1414-sd2.pdf]

Table S2

Origins of isolates typed with MLST scheme

Isolate	Country of origin	Region	Continent	ERIC type	Sequence Type	population (FST analysis)
11-8014	New Zealand		Australasia	I	1	New Zealand
12-8090	Cuba		North America	I	1	North America
12-8293	Egypt, Ethiopia		Africa	I	2	Africa
12-8296	Egypt, Ethiopia		Africa	I	2	Africa
12-8299	Egypt, Ethiopia		Africa	I	2	Africa
12-8302	Egypt, Ethiopia		Africa	I	2	Africa
12-8338	France		Europe	I	2	France/Channel Isles
12-8343	France		Europe	I	2	France/Channel Isles
p8580 (p143 2012.02.LO)	France		Europe		2	France/Channel Isles
12-8384	Georgia, Croatia		Europe	I	2	-
12-8387	Georgia, Croatia		Europe	I	2	-
12-8382	Georgia, Croatia		Europe	I	2	-
03-159	Germany	Saxony-Anhalt	Europe	I	2	Germany
01-391	Germany		Europe	I	2	Germany
12-8314	Greece	Crete	Europe	I	2	Greece
1403/1 (P8467)	Italy		Europe		2	Italy
1267/1 (P8468)	Italy		Europe		2	Italy
1388/1 (P8465)	Italy		Europe		2	Italy
UK6/10	UK	England	Europe	I	2	UK
12-8109 (4)	Isreal, Malta		mixed	I	2	-
11-8020	New Zealand		Australasia	I	3	New Zealand
11-8032	Mexico, Nicaragua, Argentina, Chile		mixed	I	4	-
12-8320	Bangladesh		Asia	I	5	Asia
11-8021	New Zealand		Australasia	I	5	New Zealand
11-8022	New Zealand		Australasia	I	5	New Zealand
11-8023	New Zealand		Australasia	I	5	New Zealand
11-8024	New Zealand		Australasia	I	5	New Zealand
11-8026	New Zealand		Australasia	I	5	New Zealand
11-8027	New Zealand		Australasia	I	5	New Zealand

11-8028	New Zealand		Australasia	I	5	New Zealand
11-8029	New Zealand		Australasia	I	5	New Zealand
11-8010	New Zealand		Australasia	I	5	New Zealand
11-8011	New Zealand		Australasia	I	5	New Zealand
11-8012	New Zealand		Australasia	I	5	New Zealand
12-8348	France		Europe	I	5	France/Channel Isles
12-8352	France		Europe	I	5	France/Channel Isles
p8581 (p106 2012.06.LO)	France		Europe		5	France/Channel Isles
12-367	Germany	Berlin	Europe	I	5	Germany
11-152	Germany	Saxony-Anhalt	Europe	I	5	Germany
09-331	Germany	Saxony-Anhalt	Europe	I	5	Germany
P7839 (Thur 99)	Germany	Thuringia	Europe	I	5	Germany
p7840 (Thur101)	Germany	Thuringia	Europe	I	5	Germany
02-360	Germany		Europe	I	5	Germany
03-019	Germany		Europe	I	5	Germany
12-8265	Greece		Europe	I	5	Greece
12-8268	Greece		Europe	I	5	Greece
12-8391	Hungary		Europe	I	5	-
AFB7	Spain		Europe		5	Spain/ Tenerife
12-8242	Switzerland		Europe	I	5	-
12-8245	Switzerland		Europe	I	5	-
12-8248	Switzerland		Europe	I	5	-
12-8251	Switzerland		Europe	I	5	-
UK1/10	UK	England	Europe	I	5	UK
UK11/6	UK	England	Europe	I	5	UK
UK15/10	UK	England	Europe	I	5	UK
UK16/8	UK	England	Europe	I	5	UK
UK17/10	UK	England	Europe	I	5	UK
UK17/9	UK	England	Europe	I	5	UK
UK3/8	UK	England	Europe	I	5	UK
UK6/8	UK	England	Europe	I	5	UK
UK7/10	UK	England	Europe	I	5	UK

UK12/10	UK	England	Europe	I	5	UK
UK14/10	UK	England	Europe	I	5	UK
UK2/10	UK	England	Europe	I	5	UK
UK2/7	UK	England	Europe	I	5	UK
UK2/9	UK	England	Europe	I	5	UK
UK20/10	UK	England	Europe	I	5	UK
UK5/9	UK	England	Europe	I	5	UK
UK7/9	UK	England	Europe	I	5	UK
UK33/9	UK	England	Europe	I	5	UK
UK38/9	UK	England	Europe	I	5	UK
UK21/8	UK	England	Europe	I	5	UK
UK22/10	UK	England	Europe	I	5	UK
UK26/7	UK	England	Europe	I	5	UK
UK24/10	UK	England	Europe	I	5	UK
UK26/9	UK	England	Europe	I	5	UK
UK27/7	UK	England	Europe	I	5	UK
UK27/9	UK	England	Europe	I	5	UK
UK35/9	UK	England	Europe	I	5	UK
UK22/11	UK	England	Europe	I	5	UK
UK21/10	UK	England	Europe	I	5	UK
UK22/9	UK	England	Europe	I	5	UK
S1/1	UK	England	Europe	I	5	UK
S2/1	UK	England	Europe	I	5	UK
12-8273	Canada		North America	I	5	North America
12-8277	Canada		North America	I	5	North America
12-8278	Canada		North America	I	5	North America
12-8281	Canada		North America	I	5	North America
12-8091 (2)	Cuba		North America	I	5	North America
p6678 (LMG 16241)	-			I	5	-
p8582	France		Europe		6	France/Channel Isles
J3/1	Jersey		Europe	I	6	France/Channel Isles
12-8378	Mongolia		Asia	I	7	Asia

12-8380	Mongolia		Asia	I	7	Asia
P8476	France		Europe		7	France/Channel Isles
UK15/6	UK	England	Europe	I	7	UK
UK20/6	UK	England	Europe	I	7	UK
UK3/6	UK	England	Europe	I	7	UK
UK10/6	UK	England	Europe	I	7	UK
UK10/7	UK	England	Europe	I	7	UK
UK21/6	UK	England	Europe	I	7	UK
UK21/7	UK	England	Europe	I	7	UK
UK23/6	UK	England	Europe	I	7	UK
UK25/6	UK	England	Europe	I	7	UK
UK34/6	UK	England	Europe	I	7	UK
UK36/6	UK	England	Europe	I	7	UK
UK37/6	UK	England	Europe	I	7	UK
UK38/6	UK	England	Europe	I	7	UK
(P6266) LMG 16252	-			III	8	-
ATCC49483	-			IV	8	-
DSM 3615	-			IV	8	-
(P6264) LMG 16247	-			IV	8	-
LMG6911	-			IV	8	-
P6993 (LMG 14427)	-			IV	8	-
P6260 (LMG 16250)	-			IV	8	-
P6265 (LMG 16249)	-			IV	8	-
11-8050	Chile		South America	III	9	South America
11-8051	Chile		South America	III	9	South America
12-8120	Arabian Peninsula		Asia	II	10	Arabian Peninsula
12-8121	Arabian Peninsula		Asia	II	10	Arabian Peninsula
12-8122	Arabian Peninsula		Asia	II	10	Arabian Peninsula
12-8123	Arabian Peninsula		Asia	II	10	Arabian Peninsula
12-8124	Arabian Peninsula		Asia	II	10	Arabian Peninsula
12-8125	Arabian Peninsula		Asia	II	10	Arabian Peninsula
12-8126	Arabian Peninsula		Asia	II	10	Arabian Peninsula

12-8127	Arabian Peninsula		Asia	II	10	Arabian Peninsula
12-8128	Arabian Peninsula		Asia	II	10	Arabian Peninsula
12-8119	Arabian Peninsula		Asia	II	10	Arabian Peninsula
12-8230	Arabian Peninsula		Asia	II	10	Arabian Peninsula
12-8231	Arabian Peninsula		Asia	II	10	Arabian Peninsula
12-8232	Arabian Peninsula		Asia	II	10	Arabian Peninsula
12-8170	Japan, Singapore, China		Asia	II	10	Asia
12-8172	Japan, Singapore, China		Asia	II	10	Asia
12-8175	Japan, Singapore, China		Asia	II	10	Asia
12-8177	Japan, Singapore, China		Asia	II	10	Asia
12-8179	Japan, Singapore, China		Asia	II	10	Asia
12-8220	Kazakhstan		Asia	II	10	Asia
12-8221	Kazakhstan		Asia	II	10	Asia
12-8222	Kazakhstan		Asia	II	10	Asia
12-8130	Australia		Australasia	II	10	Australia
12-8131	Australia		Australasia	II	10	Australia
12-8132	Australia		Australasia	II	10	Australia
12-8135	Australia		Australasia	II	10	Australia
12-8136	Australia		Australasia	II	10	Australia
12-8137	Australia		Australasia	II	10	Australia
12-8138	Australia		Australasia	II	10	Australia
12-8139	Australia		Australasia	II	10	Australia
P7860 (11-8013)	New Zealand		Australasia	II	10	New Zealand
P7846	Austria		Europe	II	10	-
03-522	Germany	Brandenburg, Kleinmachnow	Europe	II	10	Germany
03-525	Germany	Brandenburg, Kleinmachnow	Europe	II	10	Germany
02-009	Germany	SH / Bad Schwartau	Europe	II	10	Germany
p7842 (Thur 258)	Germany	Thuringia	Europe	II	10	Germany
12-109	Germany	Veitshöchheim	Europe	II	10	Germany
12-510	Germany	Veitshöchheim	Europe	II	10	Germany
01-649	Germany		Europe	II	10	Germany
00-1163	Germany		Europe	II	10	Germany

00-0869	Germany		Europe	II	10	Germany
00-897	Germany		Europe	II	10	Germany
11-8080	Greece	Mount Athos	Europe	II	10	Greece
11-8081	Greece	Mount Athos	Europe	II	10	Greece
12-8371	Russia		Europe	II	10	-
12-8369	Russia		Europe	II	10	-
12-8373	Russia		Europe	II	10	-
12-8375	Russia		Europe	II	10	-
26-02	Sweden		Europe	II	10	Scandinavia
P7371	Sweden		Europe	II	10	Scandinavia
12-8272	Canada		North America	II	10	North America
p8583 (AP1141 nr 2)	Austria		Europe		11	-
P7844 (452|03)	Czech Republic		Europe	II	11	-
8514-03	Finland		Europe	II	11	Scandinavia
8533-03	Finland		Europe	II	11	Scandinavia
P7850 (7774|03)	Finland		Europe	II	11	Scandinavia
p7852 (8527/03)	Finland		Europe	II	11	Scandinavia
02-334	Germany	Berlin	Europe	II	11	Germany
03-194	Germany	Berlin	Europe	II	11	Germany
03-200	Germany	Berlin	Europe	II	11	Germany
04-309	Germany	Berlin	Europe	II	11	Germany
05-085	Germany	Berlin	Europe	II	11	Germany
p7853 (646D2920/03)	Germany	Detmold	Europe	II	11	Germany
P7854 (647D2920|03)	Germany	Detmold	Europe	II	11	Germany
Uni_Saarl.909	Germany	Saarland	Europe	II	11	Germany
Uni_Saarl.913	Germany	Saarland	Europe	II	11	Germany
Uni_Saarl.914	Germany	Saarland	Europe	II	11	Germany
Uni_Saarl.916	Germany	Saarland	Europe	II	11	Germany
Uni_Saarl.918	Germany	Saarland	Europe	II	11	Germany
03-098	Germany	Schleswig-Holstein	Europe	II	11	Germany
10-228	Germany	Veitshöchheim	Europe	II	11	Germany
10-232	Germany	Veitshöchheim	Europe	II	11	Germany

10-654	Germany	Veitshöchheim	Europe	II	11	Germany
10-658	Germany	Veitshöchheim	Europe	II	11	Germany
10-662	Germany	Veitshöchheim	Europe	II	11	Germany
10-678	Germany	Veitshöchheim	Europe	II	11	Germany
11-365	Germany	Veitshöchheim	Europe	II	11	Germany
11-370	Germany	Veitshöchheim	Europe	II	11	Germany
11-381	Germany	Veitshöchheim	Europe	II	11	Germany
11-577	Germany	Veitshöchheim	Europe	II	11	Germany
11-593	Germany	Veitshöchheim	Europe	II	11	Germany
11-599	Germany	Veitshöchheim	Europe	II	11	Germany
11-627	Germany	Veitshöchheim	Europe	II	11	Germany
12-116	Germany	Veitshöchheim	Europe	II	11	Germany
12-128	Germany	Veitshöchheim	Europe	II	11	Germany
12-134	Germany	Veitshöchheim	Europe	II	11	Germany
12-147	Germany	Veitshöchheim	Europe	II	11	Germany
12-490	Germany	Veitshöchheim	Europe	II	11	Germany
12-498	Germany	Veitshöchheim	Europe	II	11	Germany
12-520	Germany	Veitshöchheim	Europe	II	11	Germany
11-403	Germany	Veitshöchheim	Europe	II	11	Germany
01-1714	Germany		Europe	II	11	Germany
00-0775	Germany		Europe	II	11	Germany
P7851 (8501|03)	Finland		Europe	II	12	Scandinavia
P7862 (11-8025)	New Zealand		Australasia	I	13	New Zealand
12-8262	Greece		Europe	I	13	Greece
AFB2	Spain		Europe		13	Spain/ Tenerife
J1/1	Jersey		Europe	I	14	France/Channel Isles
L1/1	South Africa		Africa	I	15	Africa
12-8133	Australia		Australasia	I	15	Australia
12-8134	Australia		Australasia	I	15	Australia
P7847	Austria		Europe	I	15	-
P7848	Austria		Europe	I	15	-
P7843 (308|03)	Czech Republic		Europe	I	15	-

01-440	Germany	Saxony-Anhalt	Europe	I	15	Germany
p7841(Thu 170)	Germany	Thuringia	Europe	I	15	Germany
01-445	Germany		Europe	I	15	Germany
01-358	Germany		Europe	I	15	Germany
03-384	Germany		Europe	I	15	Germany
782/1	Italy		Europe		15	Italy
679/1 (P8466)	Italy		Europe		15	Italy
J20/1	Jersey		Europe	I	15	France/Channel Isles
J47/1	Jersey		Europe	I	15	France/Channel Isles
12-8356	Poland		Europe	I	15	Poland
12-8361	Poland		Europe	I	15	Poland
12-8282	Tenerife		Europe	I	15	Spain/ Tenerife
12-8285	Tenerife		Europe	I	15	Spain/ Tenerife
12-8288	Tenerife		Europe	I	15	Spain/ Tenerife
12-8291	Tenerife		Europe	I	15	Spain/ Tenerife
UK1/8	UK	England	Europe	I	15	UK
UK11/10	UK	England	Europe	I	15	UK
UK13/9	UK	England	Europe	I	15	UK
UK3/10	UK	England	Europe	I	15	UK
UK23/9	UK	England	Europe	I	15	UK
UK21/11	UK	England	Europe	I	15	UK
UK1/9	UK	Wales	Europe	I	15	UK
P7858 (UK 1991)	UK		Europe	I	15	UK
11-8030	Mexico, Nicaragua, Argentina, Chile		mixed	I	15	-
11-8070	Mexico		North America	I	15	North America
12-8304	USA		North America	I	15	North America
12-8306	USA		North America	I	15	North America
12-8308	USA		North America	I	15	North America
12-8310	USA		North America	I	15	North America
USA-21	USA		North America	I	15	North America
11-8061	Argentina		South America	I	15	South America
P7864 (11-8062)	Argentina		South America	I	15	South America

B-3650	-				15	-
11-8031	Mexico, Nicaragua, Argentina, Chile		mixed	I	16	-
02-129	Germany		Europe	I	17	Germany
02-130	Germany		Europe	I	17	Germany
12-8366	Poland		Europe	I	17	Poland
P7370	Sweden		Europe	I	17	Scandinavia
P7857 (UK 1961)	UK		Europe	I	17	UK
ATCC9545	-			I	17	-
11-8040	New Zealand		Australasia	I	18	New Zealand
1487/1	Italy		Europe		18	Italy
2358/1	Italy		Europe		18	Italy
UK16/6	UK	England	Europe	I	18	UK
UK8/10	UK	England	Europe	I	18	UK
UK27/6	UK	England	Europe	I	18	UK
UK40/6	UK	England	Europe	I	18	UK
UK14/7	UK	Wales	Europe	I	18	UK
UK5/8	UK	Wales	Europe	I	18	UK
UK9/7	UK	Wales	Europe	I	18	UK
UK28/7	UK	Wales	Europe	I	18	UK
P7856 (UK 1957)	UK		Europe	I	18	UK
08-100	USA		North America	I	18	North America
USA-148	USA		North America	I	18	North America
USA-38	USA		North America	I	18	North America
P6254	-			I	18	-
12-8322	Bangladesh		Asia	I	19	Asia
12-8324	Bangladesh		Asia	I	19	Asia
12-8326	Bangladesh		Asia	I	19	Asia
p7845 (778/03)	Czech Republic		Europe	I	19	-
00-087	Germany		Europe	I	19	Germany
03-125	Germany		Europe	I	19	Germany
12-8312	Greece	Crete	Europe	I	19	Greece
12-8271	Greece		Europe	I	19	Greece

12-8355	Hungary		Europe	I	19	-
p8488	Poland	Kutno	Europe		19	Poland
p8494	Poland	Radomsko	Europe		19	Poland
P8477	Poland	Sieradz	Europe		19	Poland
P8480	Poland		Europe		19	Poland
12-8100	Isreal,Malta		mixed	I	19	-
03-189	Germany	Saxony-Anhalt	Europe	I	20	Germany
UK6/7	UK	England	Europe	I	21	UK
UK25/7	UK	England	Europe	I	21	UK
